# A Manual of Diseases of the Ear for the Use of Students and Practitioners of Medicine

**Published:** 1896-02

**Authors:** 


E. B. TREAT, Publisher, New York, has in press for early publication the 1896 International Medical Annual, being the fourteenth yearly issue of this eminently useful work. Since the first issue of this one-volume reference work, each year has witnessed marked improvements; and the prospectus of the forthcoming volume gives promise that it will surpass any of its predecessors. It will be the conjoint authorship of forty distinguished specialists, selected from the most eminent physicians and surgeons of America, England, and the Continent. It will contain reports of the progress of medical science at home and abroad, together with a large number of original articles and reviews on subjects with which the several authors are especially associated. In short, the design of the book is, while not neglecting the specialist, to bring the general practitioner into direct communication with those who are advancing the science of medicine, so he may be furnished with all that is worthy of preservation, as reliable aids in his daily work. Illustrations in black and colors will be consistently used wherever helpful in elucidating the text. Altogether, it makes a most useful, if not absolutely indispensable, investment for the medical practitioner. The price will remain the same as previous issues, $2.75.

---

DON'TS FOR CONSUMPTIVES, or the Scientific Management of Pulmonary Tuberculosis is the title of a book which, under the authorship of Dr. Charles Wilson Ingraham, will soon (about February 10th) be issued by the Medical Reporter Publishing Co., of Rochester, N. Y.

The complete work of 35 chapters is devoted exclusively to the general management of Pulmonary Invalids, no reference whatever being mode to drug treatments.

The object of the author is to supply the physician with a practical work, and at the same time, by eliminating technical terms, reduce the text within the easy comprehension of the intelligent patient. The author claims that "a good understanding of his condition is the best remedy for the consumptive." With this book in the hands of his patient the physician will be relieved of a multitude of details which attach to the successful management of such cases. Special attention has been given those chapters pertaining to the destruction of tubercular infection.

The book will be printed on 27-pound antique book paper, bound in cloth (imitation morocco), with title in gold leaf. Price, $1.75.

